# Autoimmunological serum parameters and bone mass density in premature ovarian insufficiency: a retrospective cohort study

**DOI:** 10.1007/s00404-020-05860-4

**Published:** 2020-11-09

**Authors:** Klara Beitl, Klara Rosta, Nina Poetsch, Manuel Seifried, Daniel Mayrhofer, Barbara Soliman, Rodrig Marculescu, Johannes Ott

**Affiliations:** 1 Clinical Division of Gynecological Endocrinology and Reproductive Medicine, Department of Gynecological Endocrinology and Reproductive Medicine, Medical University of Vienna, Spitalgasse 23, 1090 Vienna, Austria; 2 Department of Radiology and Nuclear Medicine, Medical University of Vienna, Spitalgasse 23, 1090 Vienna, Austria; 3 Department of Laboratory Medicine, Medical University of Vienna, Spitalgasse 23, 1090 Vienna, Austria

**Keywords:** Premature ovarian insufficiency, Bone mass density, Autoimmunity

## Abstract

**Purpose:**

It is still not clear whether to screen women with primary premature ovarian insufficiency for autoimmunity. Moreover, a possible association of autoimmunity with decreased bone mass density in premature ovarian insufficiency patients has not been evaluated. Thus, the objectives of this study were to review our experience with the use of an autoimmune screening panel in premature ovarian insufficiency women and to focus on bone mass density.

**Methods:**

In a retrospective cohort study, 76 chromosomally normal women with primary premature ovarian insufficiency were included. The main outcome parameters were the results of an autoimmune screening panel and of dual-energy X-ray absorptiometry.

**Results:**

Median age was 33 years. Sixty percent of premature ovarian insufficiency patients revealed abnormal dual-energy X-ray absorptiometry results (minimal T-score < −1.0). Any signs of autoimmunity were found in 21 women (36.2%). The most frequent abnormal results were increased thyroperoxidase antibodies (24.1%) and thyroglobulin antibodies (20.7%). A longer duration of amenorrhea (*β* = −0.015; *p* = 0.007), any abnormality during autoimmune screening (*β* = −0.940; *p* = 0.010), and a lower body mass index (*β* = −0.057; *p* = 0.036) were associated with a lower minimal T-score.

**Conclusion:**

In chromosomally normal women with primary premature ovarian insufficiency, the prevalence of autoimmunity and decreased bone mass density seem high. Our data highlight the association between autoimmune abnormalities and decreased dual-energy X-ray absorptiometry results.

## Introduction

Premature ovarian insufficiency (POI) is defined as the cessation of ovarian function before the expected age of menopause [1, 2]. In most cases, an unknown mechanism leads to irregular or absent menstrual cycles and sex steroid deficiency. It is characterized by oligo/amenorrhea for at least 4 months, and an elevated FSH level >25 IU/l on two occasions >4 weeks apart [3]. Spontaneous POI, i.e., POI without previous oncological treatment through surgery, chemotherapy or radiotherapy [1, 4], affects approximately 1–2% of women prior to age of 40 and 0.1% prior to age of 30 [1, 5].

Since a decrease in estrogen is associated with a loss of trabecular and cortical bone departments in women, patients with POI show decreased bone mineral density (BMD) of the lumbar spine and the femoral neck assessed by dual-energy X-ray absorptiometry (DEXA) [6]. About two thirds of karyotypically normal women with spontaneous POI were reported to suffer from decreased femoral neck BMD [7]. These results were confirmed by a later study: Popat et al. demonstrated that POI patients had 2–3% lower BMD at L1–L4, femoral neck, and total hip. Notably, several risk factors were identified which included but were not limited to a delayed diagnosis of estrogen deficiency, low vitamin D levels, non-adherence to estrogen replacement therapy and race [8].

In 90% of cases, the cause for spontaneous POI remains unclear [9]. However, a link between autoimmunity and spontaneous POI is very likely. Evidence that supports this hypothesis includes literature about ovarian autoantibodies, the histologically confirmed presence of lymphocytic oophoritis and the strong association of POI with other autoimmune disorders [9–11].

Notably, decreased BMD was found in patients with various autoimmune diseases, which include systemic lupus erythematosus [12], serologically suspected chronic thyroiditis [13], and autoimmune hepatitis [14]. These observations are in line with the conclusion of a recent review that autoimmunity seemed to represent an important driver in pathological bone loss [15].

Although it is widely suspected that autoimmunity was an underlying risk factor for POI, it cannot be ruled out that the decrease in sex steroids might also have an impact on autoimmunity. Levels of dehydroepiandrosterone sulfate (DHEAS) were decreased in women with POI and to be even lower in women with POI and organ-specific autoimmunity [16]. Moreover, DHEA treatment has been claimed to be beneficial in systemic lupus erythematosus [17] and possibly also in POI patients with chronic autoimmune thyroiditis [18]. Although all POI women usually benefit from sex steroid supplementation, those with signs of increased autoimmunity could benefit even more in terms of a decline in autoimmune burden, hypothetically. Thus, the association between spontaneous POI, autoimmunity, and BMD should be of special interest.

The actual prevalence of autoimmune diseases in POI remains still unclear. Moreover, it is still not obvious whether and how to screen women for autoimmunity, who suffer from primary POI. Notably, devising specific non-invasive diagnostic tools had been suggested as one of the major issues in future research on POI and autoimmunity [9]. In addition, a possible link between autoimmunity and BMD has never been evaluated in POI women. Thus, in this retrospective cohort study, we focused on the incidence of increased markers for autoimmunity that were evaluated routinely in our patient population as well as on DEXA results and serum sex steroid levels to compare BMD between POI women with and without suspicion of autoimmunity. Thereby, we wanted to contribute to the growing field of literature on this important topic.

## Materials and methods

In a retrospective case series, we included women who suffered from spontaneous POI, defined as follows: secondary amenorrhea for at least four months (in the absence of pregnancy, breastfeeding, continuous use of any medication causing amenorrhea, or surgical removal of the uterus and/or ovaries, previous chemotherapy, or pelvic radiotherapy), and elevated follicle-stimulating hormone (FSH > 25.0 IU/l) on two occasions >4 weeks apart in women under 40 years of age [3]. These women (*n* = 76) had been referred to the Clinical Division of Gynecologic Endocrinology and Reproductive Medicine from January 2015 to December 2019 and had undergone complete evaluation of hormonal parameters, autoimmune screening as described below, and dual-energy X-ray absorptiometry (DEXA). The following patients were excluded: one person with Turner syndrome and one woman with a fragile X repeat mutation. Fragile X syndrome was defined as a functional loss of FMR1 gene caused by an expansion of the CGG trinucleotide sequence in the 5′ untranslated region (5′ UTR), typically more than 200 repeats [19, 20]. Seven women had already used estrogen treatment; and in nine women, the data set was incomplete (missing data on autoimmune screening or DEXA results). This resulted in a final patient population of 58 women with spontaneous POI.

The study protocol complies with the declaration of Helsinki and was approved by the Institutional Review Board of the Medical University of Vienna (institutional review board number 2217/2019). Neither written nor verbal informed consent was necessary in retrospective studies according to the Ethics Committee of the Medical University of Vienna.

### Parameters analyzed

As main outcomes parameters, we focused on BMD at the spine and hip. DEXA was part of the clinical routine in POI patients and was performed at the Department of Radiology and Nuclear medicine of the Medical University of Vienna and a Hologic QDR 2000 TM device was used. BMD was measured at the lumbar spine (L1–L4) and the femoral neck. T-scores, which are provided in this report, are the relevant measures that reflect the number of standard deviations a patient differs from the average BMD of healthy, young subjects. According to the World Health Organization’s most recent guidelines [21], a T-score of >−1.0 is rated as normal, from −1.0 to −2.5 as osteopenia and below −2.5 as osteoporosis.

Blood samples were taken from a peripheral vein during amenorrhea. All examined blood parameters were determined at the Department of Laboratory Medicine, General Hospital of Vienna, Vienna, Austria according to ISO 9001 and ISO 15189 quality standards: estradiol, follicle-stimulating hormone (FSH), luteinizing hormone (LH), anti-Mullerian hormone (AMH) and progesterone were measured by the corresponding Cobas electrochemiluminescence immunoassays (ECLIA) on Cobas e 602 analyzers (Roche, Mannheim, Germany). In all women, a serum progesterone level <1.0 ng/mL was found and thus, it could be ruled out that ovulation had occurred. 25-hydroxy-vitamin D measurements were performed by chemiluminescence immunoassay (CLIA) with the Liaison 25 OH Vitamin D Total Assay on Liaison XL analyzers (Roche, Saluggia, Italy). HbA1c was determined on a Variant II fully automated HPLC system (BioRad Laboratories, Hercules, CA, USA). Our standard autoimmunity screening panel consisted of following parameters: thyroid autoantibodies against thyroglobulin (TGAb), thyroperoxidase (TPOAb) and the thyrotropin receptor (TRAb) (Roche Cobas ECLIA as described), total anti-nuclear antibodies (ANA, indirect immunofluorescence microscopy on Hep-2 cells), ANA subsets (against dsDNA, SSA/Ro, SSB/La, SCL-70, Sm, U1RNP, Jo-1 and centromere/CENP-B, fully automated ELISA system Phadia ImmunoCap250, Thermo Scientific, Waltham, MA, USA) and antibodies against cardiolipin and beta 2 glycoprotein 1.

The following basic patient characteristics were also included: age at evaluation, time interval between the last spontaneous menstrual bleeding and DEXA, body mass index (BMI) and presence of estrogen-deficiency-dependent symptoms (hot flushes and sleeping disturbances). These parameters were collected using AKIM® software (SAP Software Solutions Austria, Vienna, Austria).

### Statistical analysis

Categorical parameters are presented as numbers and frequencies continuous data as median and their respective interquartile range (IQR). To assess the predictive factors for the minimum T-score, a generalized linear model was used. For this model, regression coefficients beta (β) with the standard deviations are provided as well as Wald tests and the likelihood ratio tests. Statistical significance was defined by two-sided *P*-values <0.05. Statistical analyses were performed using SPSS 24.0 (IBM SPSS, USA).

## Results

The median age at evaluation of POI was 33 years (IQR, 26–36), the median BMI was 21.4 kg/m^2^ (IQR, 19.6–24.2). Patients had been amenorrhoic before DEXA evaluation for a median of 12.0 months (IQR 6.8–19.3). Twenty-nine women reported hot flushes (50.0%) and eight (13.8%) sleeping disorders. An overview on the results of POI evaluation is provided in Table 1. Thirty-seven patients (63.8%) revealed no science of autoimmunity in our panel; whereas one, two, four and six autoimmune markers were increased in 8 (38.1%), 11 (52.4%), 1 (4.8%) and 1 (4.8%) women, respectively. Notably, in the women with six increased antibodies, TPOAb, TGAb, and antibodies against cardiolipin and beta 2 glycoprotein 1 were elevated. Thus, this woman had the serologic suspicion of chronic autoimmune thyroiditis and antiphospholipid syndrome only. The table also shows that the majority of patients revealed abnormal DEXA results (*n* = 35, 60.4%). Concerning autoimmune screening, the most frequent abnormal results were increased TPOAb (24.1%) followed by increased TGAb (20.7%) and abnormal ENA subsets (5.2%). Any signs of autoimmunity were found in 21 women (36.2%).

**Table 1:** Results of basic evaluation of POI: hormonal parameters, DEXA results, and autoimmune screening

	Median (IQR)	Number (frequency) of abnormal results
Hormonal parameters		
Total estradiol (pg/mL)	14 (7; 62)	–
FSH (mIU/mL)	77.4 (39.6; 115.2)	–
LH (mIU/mL)	45.1 (26.2; 62.3)	–
AMH (ng/mL)	0.01 (0.01; 0.08)	–
25OH Vit D (nmol/L)	59.1 (40.8; 78.6)	–
DEXA results		
Lumbar spine T-score	−1.2 (−2.1; −0.5)	–
Femoral T-score	−0.8 (−1.6; −0.2)	–
Minimal T-score	−1.4 (−2.1; −0.2)	–
DEXA interpretation		
Osteopenia	–	28 (48.3)
Osteoporosis	–	7 (12.1)
Autoimmune screening		
TGAb (U/mL)	11.0 (0;18.5)	12 (20.7)^*^
* TPOAb* (U/mL)	14.0 (7.5;38.0)	14 (24.1)^*^
* TRAb* (U/mL)	0 (0;0.7)	0^*^
* ANA/ANF* (*HEp*-*2 *titer)	0 (0;0)	1 (1.7)^*,#^
ENA subsets	–	3 (5.2)^*^
Anti-*dsDNA *(IU/mL)	0 (0;0)	0^*^
Anti-*Scl-70 *(U/mL)	0.8 (0;1.0)	0^*^
Anti-Sm (U/mL)	1.0 (0.6;1.4)	1 (1.7)^*^
Anti-*U1RNP *(U/ml)	0.6 (0.3;1.0)	0^*^
Anti-*Jo-1 *(U/mL)	0 (0;0.7)	0^*^
Anti-CENP-B (U/mL)	0 (0;0.5)	0^*^
Anti-*Ro*/*SSA *(U/mL)	0 (0;0)	1 (1.7)^*^
Anti-*SSB/La *(U/mL)	0 (0;0)	0^*^
aCL IgM (U/mL)	0 (0;1.6)	2 (3.4)^*^
aCL IgG (U/mL)	1.1 (0;1.4)	2 (3.4)^*^
Anti-B2GPI IgM (U/mL)	0.6 (0;1.3)	2 (3.4)^*^
Anti-B2GPI IgG (U/mL)	0.8 (0;1.9)	2 (3.4)^*^
HbA1c (mmol)	30 (29;32)	0^*^
Any autoimmune marker positive		21 (36.2)

^*^Multiple selections possible

^#^The patient who was positive for *ANA/ANF* (*HEp*-*2 titer*) revealed a “speckled” *ANA/ANF* *Hep-2* pattern

In a generalized linear model, predictive parameters for the minimal DEXA T-score were evaluated (Table 2). A longer duration of amenorrhea before DEXA was significantly associated with a lower minimal T-score (*β* = −0.015 ± 0.0055; *p* = 0.007) as well as any abnormality during autoimmune screening (*β* = −0.940 ± 0.3659; *p* = 0.010). In contrast, a higher BMI was associated with an increased minimal T-score (*β* = 0.057 ± 0.0272; *p* = 0.036).

**Table 2:** Generalized linear model for the prediction of the minimal T-score

	Coefficient beta (standard deviation)	Wald test	*P*
Intercept	−0.270 (1.2125)	0.050	0.824
Hot flushes	0.497 (0.3423)	2.112	0.146
Sleeping disorder	0.058 (0.4687)	0.015	0.901
Positive in autoimmune screening	−0.940 (0.3659)	6.607	*0.010*
Age (years)	−0.049 (0.0255)	3.699	0.054
BMI (kg/m^2^)	0.057 (0.0272)	4.375	*0.036*
FSH (mIU/mL)	−0.006 (0.0036)	3.133	0.077
Estradiol (pg/mL)	−0.001 (0.0035)	0.028	0.866
AMH (ng/mL)	−0.322 (0.1735)	3.445	0.063
Duration of amenorrhea before DEXA (months)	−0.015 (0.0055)	7.213	*0.007*

Significant *p*-values are provided in italics

## Discussion

In this retrospective study, high rates of women with autoimmune abnormalities (36.2%) and with decreased BMD in DEXA (60.4%) were observed. Once more, these findings underline the overall importance of POI, which exceeds amenorrhea and sterility by far. Notably, POI has been claimed a “serious chronic disease with far reaching effects on physical and emotional health” [22]. The involvement of a care manager should be considered not only to ensure comprehensive care (doctors, nurses, psychologists, etc.) but also to guarantee a better overall satisfaction in terms of a collaborative team. This would improve self-management skills and, thus, patient empowerment, as well as provide the necessary advice and information. A better control of the disease and improved clinical parameters would be desirable [23].

Concerning the high rate of abnormal DEXA results (about 60%), the majority of patients showed osteopenic DEXA values (48.3% of all women), whereas osteoporotic values were found less frequently (12.1% of all women). However, it has been demonstrated that the majority of fractures occur in an osteopenic state [24]. In addition, there is evidence that low BMD predicts progression to osteoporosis later in life. Less than 10% of postmenopausal women with normal bone density or mild osteopenia develop osteoporosis within a period of 15 years, women with moderate osteopenia within 5 years, whereas women with advanced osteopenia show osteoporosis within 1 year [25]. Therefore, the result is of high clinical relevance.

In literature, the rate of POI women with an abnormal BMD differs widely. Possible explanations would be different definition criteria for abnormal DEXA results. On the other hand, bone density is probably dependent on age in POI patients. A large cross-sectional study demonstrated that among 442 POI patients, 15% had a Z-Score below the expected range of age (defined as <−2) and 8% were already in an osteoporotic state (<−2.5). The women’s median age was about 29 years [8]. In contrast, Anasti et al. reported that in karyotypically normal POI patients, 67% presented a femoral neck bone density more than one standard deviation below the mean of the reference group. This prevalence as well as the women’s median age of 33 years [7] were similar to our results. On the one hand, the cut-off criteria used to define an abnormal DEXA result might be considered of relevance. On the other hand, based on the studies cited above, one could hypothesize that the prevalence of abnormal DEXA results would increase with age. In our patient population, age closely failed to reach statistical significance (*p* = 0.054, Table 2), which might be due to the sample size.

One significant factor that was associated with a lower BMD in our data set was a longer duration of amenorrhea before DEXA measurement (Table 2). In our study patients had been amenorrhoic before DEXA evaluation for a median of 12 months. Hypothetically, amenorrhea could be seen as a sign for a more severe POI condition as well as longer lasting estrogen deficiency. In line with our results, it has already been reported that POI women who had had a 1-year delay of diagnosis after the onset of symptoms (i.e., menstrual irregularities) showed lower BMD levels than patients who were diagnosed earlier [8]. Both observations, namely the negative influence of advanced age and the delay of diagnosis on BMD, suggest that early detection seems important to establish hormone replacement therapy as soon as possible to avoid BMD decrease [26]. Furthermore, our multivariate model demonstrated a lower BMI as a risk factor for decreased BMD. It is well known that a BMI <18.5 kg/m^2^ is linked to an increased risk of fracture. Within eutrophic ranges, BMI seems to have a protective role on BMD, but this effect decreases steadily towards obesity. Previous observations indicated a nonlinear relationship between BMI and BMD [27, 28]. Notably, in our study cohort, the mean BMI was 21.4 kg/m^2^, which is in the range of normal weight.

One new and important finding is the association between the presence of autoimmune abnormalities and low bone density in women with POI. It obviously supports the assumption that autoimmunity might represent an important driver in pathogenic bone loss [15]. Decreased BMD has been found in patients with SLE [12], chronic thyroiditis [13] and autoimmune hepatitis [14] in previous studies. Although declines in BMD in patients with SLE might also be due to the SLE-related glucocorticoid treatment, several studies have pointed out that autoantibodies were able to induce osteoclast differentiation and activation and to alter bone mineral content. However, the exact mechanisms are not yet clear and deserve further exploration [15]. Moreover, it has been suggested that thyroid autoimmunity was a potential marker of higher fracture risk in patients with subclinical hypothyroidism [29, 30].

It is estimated that autoimmune disease is present in approximately 10–40% of patients with POI [9, 11]. The prevalence of about 36% found in our data set is within that range. A few studies demonstrated a prevalence of autoimmune conditions similar to our study: Doldi and colleagues [16] observed organ-specific autoantibodies in 44% of women with POI. Notably, in our study, women with genetic abnormalities had already been excluded which might explain the high rate of patients with associated autoimmunity. However, Alper et al. [31] showed evidence of autoimmune disorders in 39% of chromosomally competent POI women which is also in line with our results.

In literature, thyroid disorders proved to be most frequently associated with POI (27%), followed by Addison disease (2.5%) and diabetes mellitus (2.5%) [31–33]. However, the rates differed widely. For example, in our analysis, POI women were screened using various antibodies and the most frequently found abnormalities were elevated serum levels of TPOAb (24.1%), TGAb (20.7%) and ENA subsets (5.2%). To the best of our knowledge, only one study evaluated comparable numbers of antibodies. Zhen et al. [11] reported that women with POI had significant higher levels of PR3 and Jo-1 antibodies; whereas, thyroid microsomal antibodies, ANAs and ENA subsets were similar between POI patients and healthy controls. Nevertheless, thyroid antibodies are most often associated with POI. The highest rate had been reported by Pogacnik et al. [34] who had excluded POI patients with infectious, iatrogenic or genetic causes and found that 50% of POI women were positive for TG antibodies.

It should be noted that it seems questionable whether antibody screening in POI patients would be useful. According to the ESHRE Guideline Group on POI, screening for 21OH-Ab/ACA and TPOAb should be performed if unknown etiology or an immune disorder is suspected [3]. Since there is no possibility for non-invasive testing for the diagnosis of an autoimmune etiology, Kirshenbaum et al. [35] recommended the screening for the most common autoantibodies in women with POI, i.e,*.* steroid cell antibodies, anti-ovarian antibodies and anti-thyroid antibodies. Both studies suggested to include thyroid antibodies as part of a clinical routine screening in POI patients, which were also evaluated in our study and showed significant results.

The weakness of our study is shown by the lack of data collection concerning adrenal cell antibodies, anti-ovarian antibodies and steroid cell antibodies. Thus, important markers for autoimmune polyendocrine syndrome types I and II are missing. Notably, the parameters collected were part of an autoimmune panel available in clinical routine. Thus, we can neither provide a detailed rationale for every marker chosen, nor data on other probably important parameters. We consider this circumstance a major study limitation, although it seems worth pointing out that several other antibodies were evaluated and these rather new results should add to the knowledge about autoimmunity in POI. Moreover, the study is limited by its retrospective design and the small sample size. Due to the retrospective nature of our study, we cannot provide data on autoimmunity-related symptoms, which we consider unfortunate. Nevertheless, we did not only focus on autoimmune screening in POI patients, but also focused on DEXA findings.

## Conclusion

Our data show a high prevalence of autoimmune alterations and diminished BMD in untreated, chromosomally normal women with primary POI. Since literature lacks data on autoimmune screening in healthy young women, it cannot be finally stated, whether women with primary POI really carry a higher risk. However, autoimmunity was associated with decreased DEXA results. Thus, even if a similar autoimmune pattern would be found in a healthy population, autoimmunity might play a special role in POI. Further studies are warranted to prove these results and shed more light on the physiological surroundings and consequences of POI.

## Data Availability

None.
